# Metastatic Adenocarcinoma of the Prostate Presenting as a Penile Mass in a Patient With Acute Urinary Retention: A Case Report

**DOI:** 10.7759/cureus.90314

**Published:** 2025-08-17

**Authors:** R Koushik, Sujata Patwardhan, Supradeep Narayanaswamy

**Affiliations:** 1 Urology, Seth GS Medical College and King Edward Memorial Hospital, Mumbai, IND; 2 General Surgery, Jawaharlal Institute of Postgraduate Medical Education and Research, Puducherry, IND

**Keywords:** adenocarcinoma, adenocarcinoma penis, nkx3.1, penectomy, penile mass, penile metastasis, prostate cancer, prostrate, psa, psma pet

## Abstract

Metastatic prostate adenocarcinoma presenting as a penile lesion is extremely rare and can mimic primary penile malignancies. A 72-year-old male presented with acute urinary retention and a painless penile lesion with phimosis. The lesion had gradually enlarged over six months with intermittent bleeding. Examination revealed a 3 cm ulcerated mass on the glans near the coronal sulcus. A wedge biopsy suggested adenosquamous carcinoma. CT showed no distant metastases. The patient underwent total penectomy with perineal urethrostomy. Histopathology revealed malignancy with clear margins. Further evaluation at a major tertiary health care centre, including immunohistochemistry, confirmed metastatic prostatic adenocarcinoma (NKX3.1, PSA positive; CK7, CK20, CDX2 negative). Serum prostate-specific antigen (PSA) was elevated (37.8 ng/mL), and prostate-specific membrane antigen positron emission tomography (PSMA PET) confirmed metastasis. The patient was treated with androgen deprivation therapy (bilateral orchidectomy), abiraterone, and docetaxel.

## Introduction

Metastasis to the penis is an exceptionally rare clinical occurrence, accounting for less than 1% of all secondary malignancies involving the genitourinary tract [1]. While the most common primary sources include the bladder and rectum, prostate adenocarcinoma is a relatively uncommon origin [2]. Penile metastases often mimic primary penile cancers in presentation, leading to diagnostic challenges. We report the case of a 72-year-old man in whom initial histopathology suggested adenosquamous carcinoma of the penis; however, an elevated serum prostate-specific antigen (PSA) level, prostate-specific membrane antigen positron emission tomography (PSMA PET), and detailed slide review with additional immunohistochemistry at a tertiary oncology center confirmed metastatic prostate adenocarcinoma. This case highlights the importance of a comprehensive diagnostic approach, including immunohistochemistry and advanced imaging, especially in elderly patients with elevated PSA or risk factors for prostate cancer.

## Case presentation

A 72-year-old male presented to the emergency department with acute urinary retention. He reported a painless lesion on the glans penis that had been progressively enlarging over the past six months, accompanied by intermittent bleeding episodes occurring two to three times over the last three months. The patient had a known history of phimosis but no prior urological malignancies or relevant treatments.

**Figure 1:**
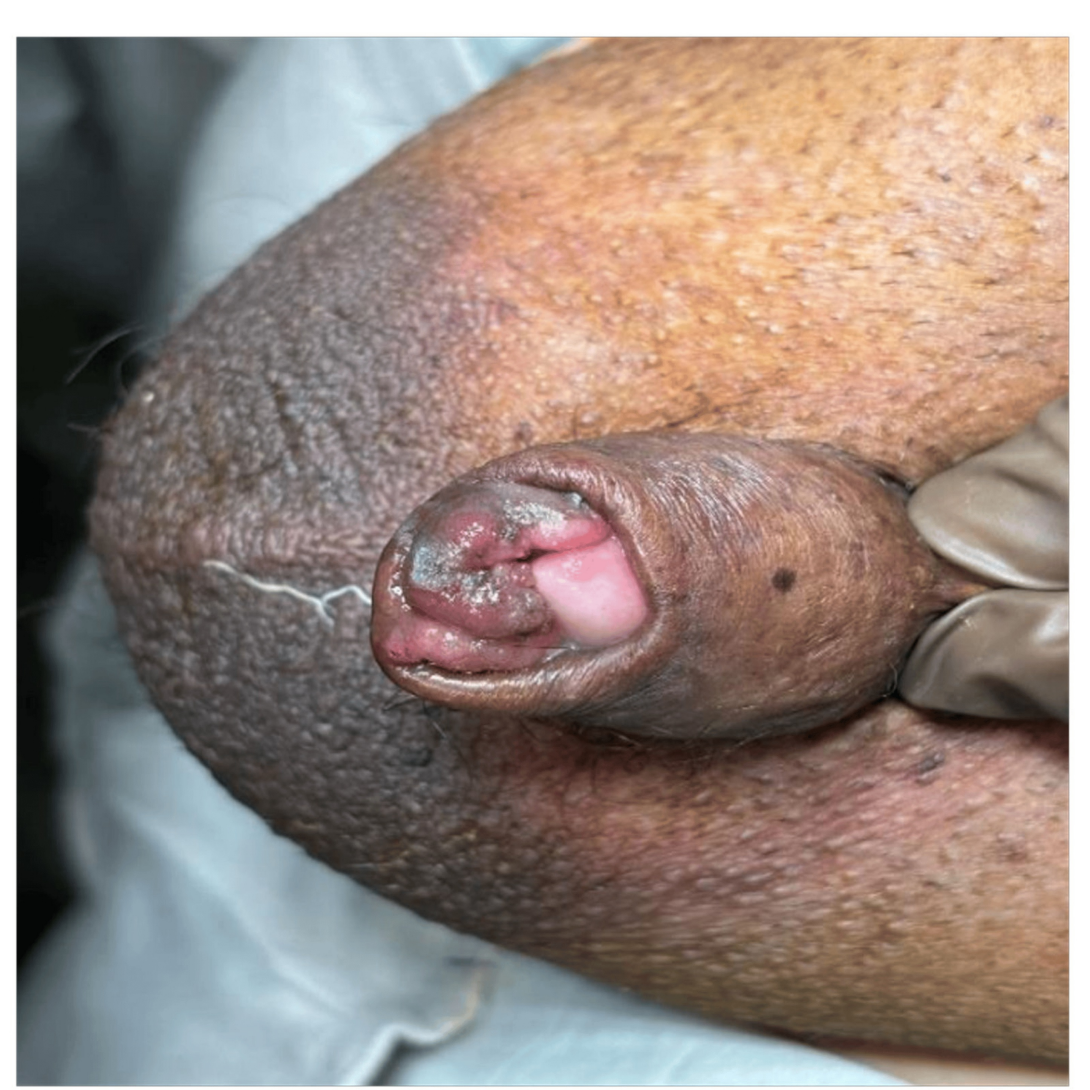
Pre-operative penile lesion

On physical examination, a 3 cm ulcerated mass with irregular borders was observed on the glans penis, extending from the coronal sulcus and involving the prepuce. Bilateral hydroceles were present, but there was no palpable inguinal lymphadenopathy. The remainder of the physical examination was unremarkable (Figure 1).

**Figure 2:**
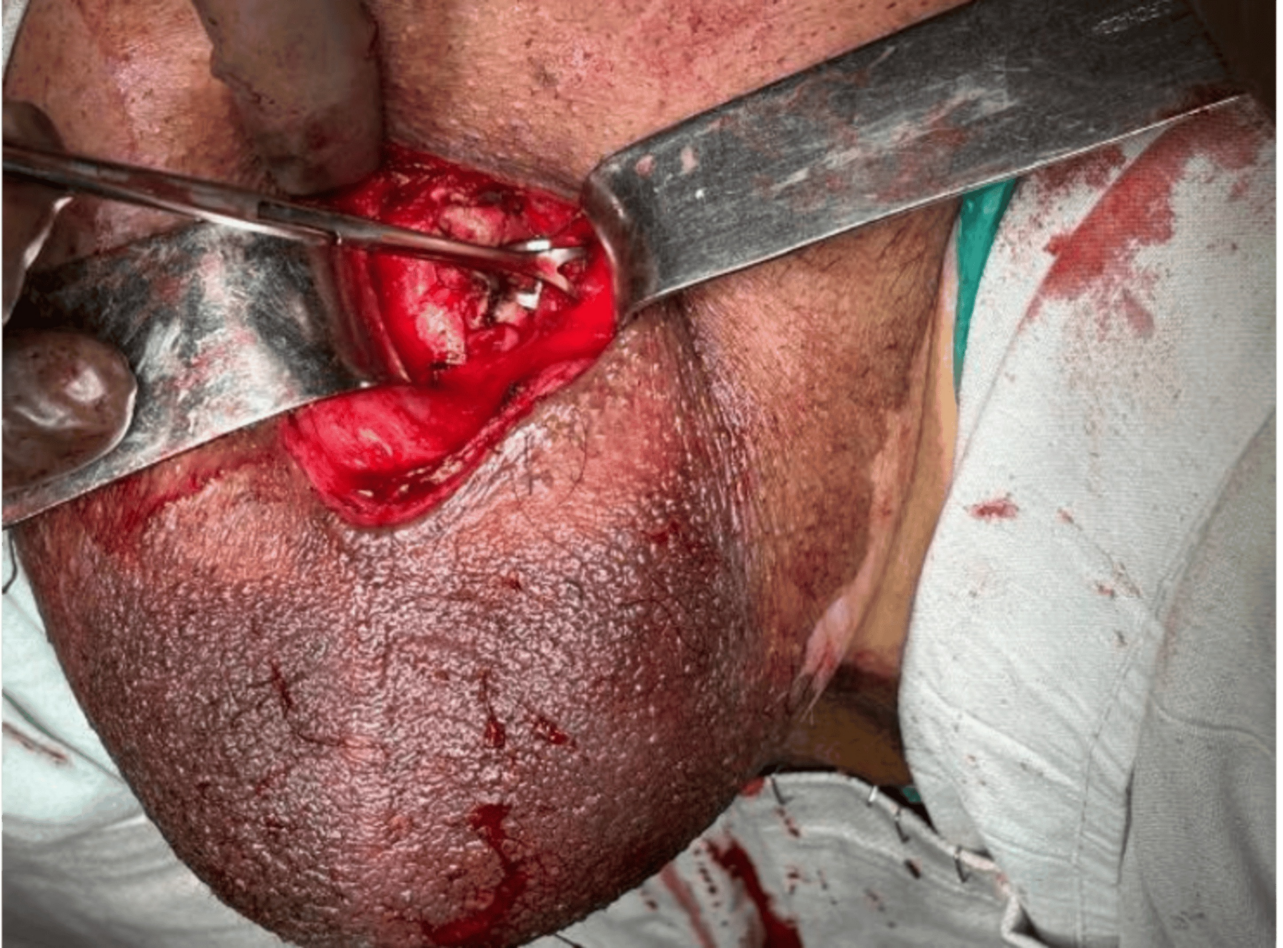
Wound after total penectomy

A wedge biopsy of the penile lesion was performed, which revealed histopathological features consistent with adenosquamous carcinoma of the penis. The patient subsequently underwent a total penectomy with permanent perineal urethrostomy (Figure 2).

Histopathological examination of the resected specimen confirmed adenosquamous carcinoma with clear surgical margins. However, further review of the slides and extended immunohistochemistry evaluation at a tertiary centre revealed a different diagnosis. The tumour cells were strongly positive for NKX3.1 and PSA, and negative for CK7, CK20, and CDX2, indicating metastatic adenocarcinoma of the prostate. Serum PSA was markedly elevated at 37.8 ng/ml (Table 1).

**Table 1:** Laboratory tests PSA: prostate-specific antigen; IHC: immunohistochemistry; GI: gastrointestinal

Laboratory test	Patient value	Normal range
Serum PSA	37.8 ng/mL	0–4.0 ng/mL
NKX3.1 (IHC marker)	Strongly positive	Not detected in non-prostatic tissues
PSA (IHC marker)	Strongly positive	Negative/weak in non-prostate tissues
CK7 (Cytokeratin 7)	Negative	Often positive in many adenocarcinomas
CK20 (Cytokeratin 20)	Negative	Often positive in colorectal/GI malignancy
CDX2	Negative	Positive in colorectal origin

A PSMA PET scan confirmed the diagnosis, showing an enlarged prostate with diffuse intermediate-grade PSMA uptake (SUVmax: 7.31) and preserved fat planes with the rectum. Bilateral seminal vesicles appeared normal. 

Low-grade PSMA uptake was noted in the bilateral internal iliac lymph nodes, with the largest node on the left measuring 1.8 × 0.9 cm (SUVmax: 4.85). Intermediate-grade PSMA expression was also seen in bilateral subcentimetric superficial inguinal lymph nodes (SUVmax: 8.72) and para-aortic lymph nodes, with a reference node on the left measuring 1.2 × 0.85 cm (SUVmax: 5.96). Additionally, multiple sclerotic lesions, more than lytic bone lesions, demonstrated PSMA expression, including involvement of the 1st and 4th right ribs and 3rd left rib.

In light of the revised diagnosis, the treatment plan was altered. Bilateral superficial inguinal lymph node dissection was not performed. Instead, the patient was managed as a case of metastatic adenocarcinoma of the prostate. He underwent bilateral orchidectomy for androgen deprivation therapy (ADT) and was started on abiraterone and docetaxel chemotherapy.

Postoperatively, the patient recovered well and is currently on abiraterone, zoledronate, and docetaxel, with ongoing oncological follow-up. He tolerated the treatment well without significant adverse effects and demonstrated good clinical response, with resolution of symptoms and no evidence of local recurrence or new metastatic lesions.

## Discussion

Penile metastasis is a rare and often unexpected clinical finding, typically indicating advanced-stage malignancy elsewhere in the body. Due to its rarity and clinical overlap with primary penile cancers, metastatic disease to the penis is frequently misdiagnosed. Clinical presentations of penile metastasis are highly variable and may include nodules or masses on the penis, with or without ulceration. Patients can also exhibit obstructive or irritative urinary symptoms, hematuria, or even priapism [3].

In this case, the patient presented with a painless ulcerative lesion on the glans penis, which was initially diagnosed as adenosquamous carcinoma on wedge biopsy. This diagnosis, although rare, is a recognized form of primary penile malignancy. However, the atypical presentation and elevated serum PSA levels warranted further investigation.

According to Abehouse, several mechanisms have been proposed to explain the spread of prostate adenocarcinoma to the penis. These include direct extension, implantation, hematogenous spread, and lymphatic dissemination [4]. Histopathology alone may be insufficient to distinguish between these entities, particularly when the morphology overlaps. Hence, immunohistochemical profiling and advanced imaging, such as PSMA PET, play a pivotal role in establishing the correct diagnosis.

The outlook for patients with penile metastasis is generally unfavourable. In a Japanese study, it was observed that 71% of patients succumbed to the disease within six months following their diagnosis [5,6].

Ghosh et al. reported that histopathological and immunohistochemical correlation between primary and metastatic sites using prostate core biopsy, penile lesion biopsy, and lymph node cytology is essential for confirming diagnosis [7]. Laert et al. noted that MRI, though sometimes nonspecific, is valuable for assessing local extent, including involvement of corpora cavernosa/spongiosum and adjacent tissues [2]. Chau et al. highlighted the role of PSMA PET in early detection of metastatic deposits with high sensitivity [8]. While these modalities provide valuable diagnostic guidance, a universal algorithm is difficult to establish, as management must be individualized based on patient presentation, comorbidities, and extent of disease.

This case highlights the importance of maintaining a high index of suspicion for secondary malignancy in older males presenting with penile lesions, especially in the context of elevated PSA or other signs suggestive of prostate cancer. Accurate diagnosis not only prevents unnecessary surgical interventions but also ensures timely initiation of appropriate systemic therapy.

Learning points

Penile lesions in elderly males, especially those with elevated PSA, should prompt consideration of metastatic prostate cancer. Additionally, the PSMA PET scan is a valuable tool in identifying and confirming metastatic prostate adenocarcinoma. Comprehensive histopathological evaluation, including immunohistochemistry, is essential in distinguishing primary penile malignancies from metastatic lesions.

## Conclusions

This case highlights the need to consider metastatic prostate adenocarcinoma in elderly males with penile lesions and elevated PSA. PSMA PET imaging and immunohistochemistry are vital for accurate diagnosis. Timely recognition allowed appropriate management with ADT, abiraterone, and docetaxel.
